# Acute Epstein - Barr virus hepatitis without mononucleosis syndrome: a case report 

**Published:** 2017

**Authors:** Afshin Moniri, Payam Tabarsi, Majid Marjani, Zahra Doosti

**Affiliations:** 1 *Virology Research Center (VRC), National Research Institute of Tuberculosis and Lung Diseases (NRITLD), Shahid Beheshti University of Medical Sciences, Tehran, Iran.*; 2 *Clinical Tuberculosis and Epidemiology Research Center (CTERC), National Research Institute of Tuberculosis and Lung Diseases (NRITLD), Shahid Beheshti University of Medical Sciences, Tehran, Iran*; 3 *Infectious Diseases and Tropical Medicine Research Center (IDTMRC), Shahid Beheshti University of Medical Sciences, Tehran, Iran*

**Keywords:** Epstein-Barr virus, Hepatitis, Infectious mononucleosis syndrome

## Abstract

Elevated liver enzymes accompanied by Infectious Mononucleosis syndrome are widely seen in primary Epstein-Barr virus infection while acute symptomatic hepatitis without typical presentations of EBV is extremely rare. In the following report, we present a patient with acute isolated hepatitis due to laboratory confirmed Epstein-Barr virus.

## Introduction

 Epstein-Barr virus (EBV) is a gamma herpes virus which infects humans around the world with high prevalence. The infection is mostly asymptomatic in children under 5 years, but it causes infectious mononucleosis syndrome in young adults characterized by fever, tonsillitis (exudative or non-exudative) and lymphadenopathy. Known presentations and complications include: prolonged malaise, fatigue, nausea, vomiting, anorexia, splenomegaly and maculopapular generalized skin rash. Abnormal liver function tests are common but self-resolving in primary infection (1, 2). Acute symptomatic hepatitis in the course of Infectious Mononucleosis syndrome is rare, and this manifestation without mononucleosis syndrome, is extremely rare. Here, we report a patient with isolated Epstein-Barr virus hepatitis.

## Case Report

A 23-year-old female referred to Masih Daneshvari hospital, the tertiary referral center of infectious disease, Tehran, Iran, with chief complaints of fever, anorexia, nausea, vomiting, mild abdominal pain and jaundice since 3 days ago. She mentioned an episode of coryza and malaise one week before, which lasted for about 4 days and resolved without any medication. She had a history of uncomplicated caesarean section 6 months ago, and her baby was alive and healthy. She did not have a history of consumption of drugs and alcohol or travel to areas with high endemicity for any infectious diseases during the last 6 months.

Her general condition was bad with yellowish discoloration of sclera and skin. On physical examination, she had tachycardia (110 beats/min) and oral temperature was 38.5 degrees centigrade. Mild right upper quadrant tenderness was present without Murphy's sign. The span of liver and spleen were 110 mm on midclavicular line and 170 mm, respectively. Cervical lymphadenopathy, exudative tonsillitis, buccal mucosa exanthema and skin rash were not detected on examination. 

Ultrasonography revealed the liver within the normal size limits, but with increased echogenicity (grade 2 fatty liver). Spleen had been enlarged with 175 mm length. Portal vein diameter was 10 mm with normal flow, and suprahepatic inferior vena cava was normal. Gallbladder and biliary tree were normal, without any signs of cholestasis. Mild ascites was present in the abdominal cavity.

Primary lab data revealed WBC: 16300 (Lymphocyte: 57%, lymphocyte variant: 20%, PMN: 4%, monocyte: 16%, others: 3%), hemoglobin: 9.4g/dL, Plt: 74000, Cr: 0.9 mg/dL, PT: 12.3, INR: 1.11, PTT: 40, AST: 239 IU/L, ALT: 219 IU/L, AlkPh: 1294 IU/L, Bilirubin (total): 15.9 mg/dL, Bilirubin (direct): 8.9 mg/dL, Albumin: 2.4 g/dL, Amylase: 19 IU/L (normal), Uric acid: 3.6 mg/dL, Triglyceride: 293 mg/dL, Ca: 7.4 mg/dL, P: 4.8 mg/dL, ESR:21, CRP: 39, Blood Cultures (twice): negative. With due attention to the prior tests which had revealed hepatitis, seeking for the cause started. Serologic tests for viral hepatitis were as follows: HBsAg: negative, Anti HBS Ab: 1, Anti HBC Ab (total): non-reactive, Anti HBC Ab (IgM): non-reactive, HBV-PCR: negative, Anti HCV Ab: reactive, HCV-RNA (RT-PCR): negative, Anti HAV Ab (total): 13.6 (within normal range), Anti HEV Ab: non-reactive, Anti HIV (1, 2) Ab: non-reactive, CMV-PCR: negative, Viral Capsid Antigen (VCA) Ab for EBV (IgM): 32 (positive). Considering the serologic results, EBV-PCR was requested that indicated positive (viral load: 151,000 IU/mL). The results of investigations for an autoimmune causes revealed ANA: negative, ASMA: 1/160 (positive), anti LKM Ab: negative. Complementary tests were Ceruloplasmin: 0.20 (normal), serum protein electrophoresis: normal, Urine Analysis: Amber colored, turbid appearance, urobilinogen (3+), bilirubin (1+), protein (1+), otherwise normal.

The patient was closely observed for probable fulminant hepatic failure during her hospital stay. Her general condition improved significantly during that time. Fever, nausea, vomiting and malaise ceased and her appetite became normal. Finally, she was discharged under good general conditions and normal liver function tests, and was asked to refer one month later for general assessments and future evaluations for autoimmune and HCV hepatitis.

During six months of follow-up, all hematologic and liver function tests were normal, and the EBV viral load become negative.

## Discussion

Epstein-Barr virus, a gamma herpes virus with more than 80% seroprevalence, is one of the most common infections all over the world. About 50% of children under 5 years of age become seropositive, from close contact that involve exchange of oral secretions via shared items such as toys, bottles, and utensils; in these children, most infections are asymptomatic or produce an acute illness that is often not recognized as being due to EBV. The rest converts mostly in young adulthood with presentation of EBV infectious mononucleosis characterized by fever, tonsillitis (exudative or non-exudative) and lymphadenopathy. Other common manifestations are prolonged malaise, fatigue, nausea, vomiting and anorexia. On physical examination, splenomegaly and maculopapular generalized rash may be present. Jaundice develops in less than 10% of young adults and 30% of elderly affected individuals. Less common complications include psychological and respiratory problems, meningitis, encephalitis, Guillain-Barre syndrome, aplastic and auto-immune hemolytic anemia, splenic rupture, myocarditis and hepatic necrosis (1-3). Abnormal liver function tests, especially elevated transaminases and hyperbilirubinemia, are common in primary infection occurring particularly in adult populations and EBV is known as an important cause of cholestasis in those patients; nevertheless, considering some reports which have shown EBV induced cholestatic hepatitis in children, it is invaluable to emphasize that Epstein-Barr virus infection should be part of the differential diagnoses in all age groups (4, 5). In uncomplicated EBV infectious mononucleosis syndrome, the rates of mortality and morbidity are very low. There is no effective antiviral therapy available for EBV infectious mononucleosis in immunocompetent persons. Acyclovir and ganciclovir are used to reduce EBV shedding but are clinically ineffective. In one study, complicated EBV infection, associated with both fulminant hepatic failure and auto-immune hemolytic anemia, was managed successfully by plasmapheresis, acyclovir and prednisolone, but there is no convincing evidence to warrant their clinical use in uncomplicated cases (6).

To the best of our knowledge, abnormal liver function tests without symptomatic hepatitis are widely seen in acute EBV infection although self-limiting, and acute symptomatic hepatitis is rare (7). On the other hand, some reports and studies have revealed the coincidence of acute hepatitis and IM syndrome due to primary EBV infection (8-10), and one presented fatal hepatitis due to reactivation of Epstein-Barr virus (11).

Some reports and review articles have revealed the relationship between EBV and autoimmune liver disease and suggested EBV to be a trigger agent for autoimmune hepatitis; therefore, chronic liver disease might be a manifestation of chronic EBV infection with frequent reactivations and persistent, moderate or low levels of viral load (12, 13).

Thus, to the best of our knowledge, isolated EBV hepatitis and cholestasis without IM syndrome is a very rare manifestation of primary EBV infection and only a few reports have introduced cases with acute (fulminant) hepatitis without IM syndrome (14). According to these reports and ours, EBV induced isolated hepatitis should be considered in all patients presenting with typical symptoms and signs of acute hepatitis, especially significant hyperbilirubinemia and cholestatic hepatitis. Furthermore, the data provide evidence for the need to test the presence of EBV in cryptogenic hepatitis even if it remains difficult to prove a causative role for EBV (15).

Considering the wide range of viral causes of acute hepatitis, after exclusion of common viral agents, EBV should be considered as an important factor

## Conflict of interest

The authors declare that they have no conflict of interest.
